# Streptococcal infection and its antimicrobial resistance profile associated with bovine mastitis in Ethiopia: a systematic review and meta-analysis

**DOI:** 10.3389/fvets.2025.1503904

**Published:** 2025-03-12

**Authors:** Melkie Dagnaw Fenta, Melaku Getahun Feleke, Atsede Solomon Mebratu, Bemrew Admassu Mengistu, Yitayew Demessie

**Affiliations:** ^1^Department of Veterinary Clinical Medicine, College of Veterinary Medicine and Animal Science, University of Gondar, Gondar, Ethiopia; ^2^Department of Veterinary Pharmacy, College of Veterinary Medicine and Animal Science, University of Gondar, Gondar, Ethiopia; ^3^Department of Biomedical Sciences, College of Veterinary Medicine and Animal Science, University of Gondar, Gondar, Ethiopia

**Keywords:** antimicrobial resistance, bovine mastitis, meta-analysis, prevalence, *Streptococcus*

## Abstract

**Background:**

In Ethiopia, bovine mastitis is a major problem affecting production, welfare, and public health. *Streptococcus* is a key pathogen that causes mastitis and is often treated with antimicrobials, which can lead to antimicrobial resistance. Nevertheless, the administration of antimicrobials can unintentionally facilitate the emergence of antimicrobial resistance. Thus, this study aimed to systematically review and estimate the pooled prevalence of streptococcal infection in bovine mastitis in Ethiopia, along with associated antimicrobial resistance profiles, to provide a comprehensive understanding of the current situation and guide effective treatment this bacteria.

**Methods:**

This systematic review was carried out according to the PRISMA guidelines. To estimate the pooled proportion and resistance, a random effects model was utilized with R software. The databases used included SCOPUS, PubMed, HINARI, Web of Science, Google, and Google Scholar.

**Results:**

Twenty-five articles were included in this meta-analysis. The overall pooled proportion of mastitis associated with *Streptococcus* spp. was 20% (95% CI: 17–23%). Significant heterogeneity was observed in the studies included (*I^2^* = 87%; *p* < 0.01). Among the regions, the highest proportion was reported for South Nation, Nationality of Peoples Region (SNNPR) at 26%, followed by Amhara (24%), Oromia and Addis Abeba (19%), and Tigray (15%). The highest proportion of *Streptococcus* isolates was found in patients with clinical mastitis (24%). Among the major *Streptococcus* spp., *Str. agalactiae* had the highest pooled prevalence at 13%. The greatest prevalence of resistant *Streptococcus* was observed against penicillin (52%), followed by streptomycin, tetracycline, and ampicillin (42, 38, and 35%, respectively). According to the information provided by this meta-analysis, evidence-based risk management measures should be established to prevent and control streptococcal infection in dairy cattle. Monitoring and reporting of streptococcal mastitis and antimicrobial resistance are needed in Ethiopia’s different regions. To minimize resistance, stricter guidelines should be implemented for antimicrobial use in dairy cattle, with a particular focus on reducing penicillin use.

## Introduction

Ethiopia has the largest livestock population in Africa, with an estimated total of approximately 70 million cattle. Cows constitute 55.9% of the country’s cattle population, with approximately 20.7% of the entire cattle population being composed of milking cows (1). The milk derived from dairy cows serves as an essential dietary resource for the majority of the urban and peri-urban population (2). A total of 85–89% of the overall national milk production is attributed to cattle (3). However, the quantity of milk falls significantly below the national demand owing to various factors that contribute to diminished milk production (4). Bovine mastitis is a major and serious disorder that has a significant effect on dairy production and is a high public health threat. It causes substantial economic losses due to reduced milk yield, treatment costs, the discarding of milk with antimicrobials, the lower price of poor-quality milk, and death from severe inflammation (5). The estimated economic losses associated with clinical mastitis are between $69 and $110 per cow on farms worldwide (6).

Mastitis can be classified by clinical signs, duration, and epidemiology. Clinical mastitis ranges from mild udder infection to severe systemic illness, with approximately 10% of cases resulting in mortality (7, 8). It presents with rapid onset, swelling, and redness of the affected quarter. In contrast, subclinical mastitis often remains undiagnosed because of the absence of visible changes in milk (9). In terms of duration, mastitis can be acute, sudden, or chronic and is characterized by a prolonged inflammatory process and the gradual development of fibrous tissue (10). Epidemiologically, mastitis can be classified into environmental and contagious forms, each caused by various agents (11). Globally, bovine mastitis affects 30 to 50% of cows annually (6).

Mastitis is caused by various pathogens, including bacteria, fungi, and viruses, with approximately 150 agents identified, with bacteria being the most common (12). In bovine mastitis, *Staphylococcus*, *Streptococcus*, and coliform bacteria are particularly harmful to the udder (13). *Staphylococcus* and *Streptococcu*s are responsible for 85–95% of bovine mastitis cases (14, 15). *Streptococcus* accounts for 10–30% of cow mastitis cases (16). The pathogenicity of *Streptococcus* is reliant on its capacity to transfer (or acquire) a range of virulence factors through gene exchange (17). *Streptococcus* demonstrates proportions strong adsorption and ant phagocytic activity. Its virulence factors include neuraminidase, lipoteichoic acid, capsular polysaccharide antigen, pyrogenic exotoxin, M protein, CAMP factor, and hemolysin (18, 19). Different virulence factors are linked to specific genetic markers, such as the *α*-antigen and *β*-antigen, which are encoded by the *bac* and *bca* genes (20). *Str. agalactiae* is a leading cause of bovine mastitis and has significant economic impacts. It can persist in bovine mammary glands by forming biofilms and is strongly linked to subclinical mastitis (17). Typically, *Str. agalactiae* is beta-hemolytic and is responsible for most mastitis infections in Africa (49%) and Asia (40%).

Several epidemiological studies have examined streptococcal infections in dairy cattle in Ethiopia. *Streptococcus* occurrence in clinical and subclinical bovine mastitis ranges from 6% (21) to 37% (22). The prevalence of bovine mastitis associated with streptococcal infection varies between 1 and 26% at the species level (23, 24), with *Str. uberis* and *Str. agalactiae* being the most commonly identified isolates. Additionally, a study (25) *reported the presence of Str. agalactiae* in 10.3% of mastitis milk samples in the Haramaya district of eastern Ethiopia.

Antimicrobial agents are the primary treatment for bacterially induced bovine mastitis in most African countries, including Ethiopia, despite increasing antimicrobial resistance (AMR) globally (26, 27). If unchecked, AMR could cause more than 10 million deaths annually by 2050 and cost more than $100 billion (28). Common antimicrobials in Ethiopia include penicillin, sulphonamide, ampicillin, cloxacillin, oxy-tetracycline, penicillin-procaine, streptomycin, and intra-mammary ampicillin-cloxacillin combinations (22). The regulation of antimicrobial utilization and veterinary practices in livestock production plays a critical role in addressing mastitis, a prevalent and economically significant disease in dairy cattle. Examining the legal framework surrounding anti-mastitis therapy is particularly important in Ethiopia, where challenges such as limited access to veterinary care, inadequate enforcement of antimicrobial regulations, and unregulated drug distribution impact treatment choices (29, 30). Ethiopia has national guidelines on antimicrobial use in livestock, but enforcement remains inconsistent, leading to the misuse of antibiotics and the emergence of antimicrobial resistance (AMR) (31, 32).

*Streptococcus* resistance can be phenotypic or genotypic, with genes such as *tet*(M) and *tet*(O) for tetracycline resistance and *erm* for macrolide resistance (33–35). The cure proportions for mastitis vary from 64 to 91% (36) and are influenced by pathogen resistance and virulence (37). In Ethiopia, resistance is high: 20% for *Str. agalactiae*, 40% for *Str. dysgalactiae*, and 33.3% for *Str. uberis* to penicillin; 40% for *Str. agalactiae* and 42.9% for *Str. uberis* to ampicillin (25, 38); 73.3% for *Str. dysgalactiae* to oxy-tetracycline; and 50% for *Str. agalactiae* to streptomycin (22, 39). The growing concern over Antimicrobial resistance further complicates this issue, as it limits the effectiveness of conventional treatments, leading to persistent infections and increased transmission risks. A survey in Ethiopia revealed that 31.8% of individuals consume raw milk (40), indicating health risks, as raw milk supports microorganism growth. *Streptococcus* spp. can cause severe human infections (41). The Ethiopian dairy sector is growing, with efforts to increase productivity and address animal diseases through epidemiological data. However, raw milk consumption and inadequate hygiene practices are concerns (42, 43). A One Health approach is essential for managing AMR and ensuring health outcomes. This review was prompted by repeated mastitis cases at the University of Gondar Veterinary Dairy Farm, in which *Streptococcus* spp. were isolated in 45% of 20 mastitis cases, 6 (30%) with *Staphylococcus*, 2 (20%) with *E. coli*, and 3 (10%) were unidentified. Therefore, this systematic review and meta-analysis aimed to estimate the pooled prevalence of streptococcal infections in bovine mastitis patients and their antimicrobial resistance profiles in Ethiopia. The findings will offer evidence-based recommendations for improved management practices, which are essential for enhancing dairy production, safeguarding animal health, and ensuring the sustainability of Ethiopia’s dairy industry. Additionally, this research will advance the understanding of the epidemiology of these infections, underscore the need for targeted interventions, and support the development of effective treatment protocols and monitoring systems for responsible antimicrobial use in veterinary medicine.

## Methods

### Search strategy

The literature review was conducted from January 12–20, 2024, using the PRISMA checklist (44). This systematic evaluation of *Streptococcus* spp. in bovine mastitis and antimicrobial resistance involved seven key stages: suitability assessment, information sources, search strategy, outcome variables, data extraction, study quality evaluation, and data synthesis with statistical analysis. A comprehensive search was conducted using several databases, including PMC, SCOPUS, PubMed, HINARI, Web of Science, Google, and Google Scholar. Study selection was performed independently by two authors (M.D.F and A.S.M). The research question addressed the proportion, prevalence, and antimicrobial resistance of *Streptococcus* spp. causing bovine mastitis in Ethiopia. Meanwhile, the key words used were *Streptococcus* spp. OR *Streptococcus* infection, epidemiology OR prevalence OR infection proportion, cattle OR bovine OR animals, resistance proportion OR antimicrobial resistance AND (mastitis) AND Ethiopia. A restriction was placed on the language of publication as English. All identified studies were imported into EndNote 20 software to remove duplicates and citations of the references.

### Description of the study settings

The meta-analysis was conducted in Ethiopia, which is located in the Horn of Africa between 3°00′–15°00′ N latitude and 32°30′–48°00′ E longitude. Covering 1.04 million square kilometers, Ethiopia is Africa’s second most populous country, with 123 million people. The country supports significant agricultural production, with approximately 70 million cattle, 52.5 million sheep, and 42.9 million goats (1). Its diverse topography includes highlands above 2,300 m, a. s.l. proportion transition zone between 1,500 and 2,300 m, and lowlands below 1,500 m.

## Study eligibility criteria

### Inclusion criteria

To avoid reviewer bias, the search was carried out by three subject matter experts in veterinary clinical medicine, veterinary pharmacy, and veterinary public health and epidemiology. All of the primary descriptive studies that had been published in English and that showed the presence of *Streptococcus* spp. in dairy cattle were included in this meta-analysis. Articles that provided a precise estimate of the percentage of each bacterial isolate were required to meet the inclusion criteria. The research needed to come from observational studies, and the cause of bacterial mastitis in cows had to be determined from clinical or subclinical cases. The study animals were limited to domestic cattle, or cows, which are commonly raised for their milk. It was necessary to gather samples from animals that had not been exposed to an experimental infection. The geographical location of the bacterial isolates had to be Ethiopia, and the isolates were identified at least down to the genus level. The overall quantity of *Streptococcus* spp. investigated and the quantity of isolates that were resistant or sensitive may or may not have been disclosed. In cases where the scientific papers presented findings from identical sample times and methodologies but with varying *Streptococcus* spp., each occurrence was documented as an individual investigation within our database. Consequently, one scientific article could encompass multiple studies.

### Exclusion criteria

Studies focusing on milk from camels and other non-cattle species were excluded from the analysis. Additionally, studies that failed to provide clear and comprehensive estimates of the proportion of each bacterial species in relation to the affected host were not included. Review articles, duplicate studies, publications containing only abstracts, qualitative research, and studies based solely on KAP (knowledge, attitudes, and practices) questionnaires, book chapters, case reports, editorials, short communications, opinion pieces, and studies without original data were excluded. Furthermore, intervention studies that did not include baseline data on the association between animal exposure and disease were excluded from the meta-analysis.

### Definition of outcome variable

In this review, we have two outcome variables: first, the pooled proportion/magnitude of *Streptococcus* spp. among the bacteria causing mastitis, and second, the antimicrobial resistance (AMR) profile of *Streptococcus* spp. In the first case, the proportion of mastitis-associated *Streptococcus* infection was estimated by considering the number of *Streptococcus* spp. isolates in the milk sample relative to the total number of bacterial isolates. In the second case, the resistance proportion of mastitis-associated *Streptococcus* isolates was calculated by determining the number of AMR isolates of *Streptococcus* spp. relative to the total number of isolates.

### Data extraction

Two investigators (B.A.M and M.G.) extracted the data independently. Data extraction, both quantitative and qualitative, was performed via two tables and an Excel spread sheet from the included studies. The primary author’s name, the year the work was published, the region, the total number of bacterial isolates, the number of isolates of *Streptococcus* spp. (the main outcome of interest), diagnostic procedures, data collection, and ethical considerations were included in the extracted components. Information was extracted from each article and entered into a database, including the antimicrobial susceptibility testing methodology (disc diffusion or minimum inhibitory concentration (MIC) estimation), MIC methodology (broth dilution method, agar dilution method, or other), number of *Streptococcus* isolates analyzed, number of resistant isolates, and type of mastitis (clinical or subclinical). Conflicts were settled through discussion and advice from a third author.

### Study quality assessment

To confirm the review’s methodological quality, a quality assessment was carried out by two independent authors (Y.D and A.S.M). The AXIS quality tool (45) was used to evaluate the included studies’ quality. The study design, sample size justification, sample representativeness, target population, use of validated measures, diagnosis of statistical methods, sample selection, sample frame, and discussion of nonresponse bias, funding reporting, and conflicts of interest are just a few of the items included in this quality assessment tool.

### Data synthesis and statistical analysis

R software was used to perform a meta-analysis via the “metaprop” function from the “meta” package version 4.1. 3–0 (46) and “metafor” in R Studio (47). The pooled proportion and 95% confidence intervals (CIs) were estimated via a random effects model based on the restricted maximum likelihood (REML) method, which computes within-and between-study variability. It was applied to the resistance proportion, heterogeneity, overall effect size, and weight of each study in the meta-analysis. Furthermore, the pooled prevalence and resistance of bovine mastitis associated with streptococcal infection are illustrated via graphs and tables. The resulting variable is binary (i.e., the only parameter available to measure effect size for single groups (e.g., *Streptococcus* positive or negative); resistant/sensitive to the antimicrobial agent) was the raw proportion with 95% confidence intervals (48). In accordance with (49), a logistic-normal random-effect regression model was used to estimate pooled proportions via logit transformation, whereas a mixed effect logistic regression model was employed for subgroup analysis.

### Investigation of heterogeneity

The sources of heterogeneity were evaluated via the Cochran’s Q test (reported as the *p* value), τ^2^ (variance between studies), and the inverse variance index (I^2^), which indicates the proportion of total variation observed between studies as opposed to heterogeneity as a result of chance. According to (50), the I^2^ index was calculated to correspond to values of 25, 50, and 75%, respectively, and was estimated to represent low, moderate and high heterogeneity. When the Q test produced a *p* value of less than 0.10 and the I^2^ value was greater than 50%, heterogeneity was deemed statistically significant. A forest plot was used to evaluate the level of heterogeneity among the studies. Each study’s weights, effect sizes, and 95% confidence intervals are displayed in a forest plot diagram.

### Subgroup sets

A subgroup analysis of the proportion of *Streptococcus* spp. in bovine mastitis was carried out on the basis of the study year, study location or region, species of bacteria, and level of mastitis (clinical and subclinical) to ascertain specific between-study variability.

### Publication bias assessment

Publication bias is typically assessed via Egger’s test, Begg’s rank test, and a funnel plot, which allows for the visual assessment of asymmetry (48). Therefore, Egger’s regression test and funnel plot diagrams were used to evaluate publication bias.

### Sensitivity and influential analysis

A sensitivity analysis of the studies was performed to evaluate the effect of each individual study (51). The results revealed that the studies were the prime determinants of the pooled result.

## Results

### Search results

**Figure 1:**
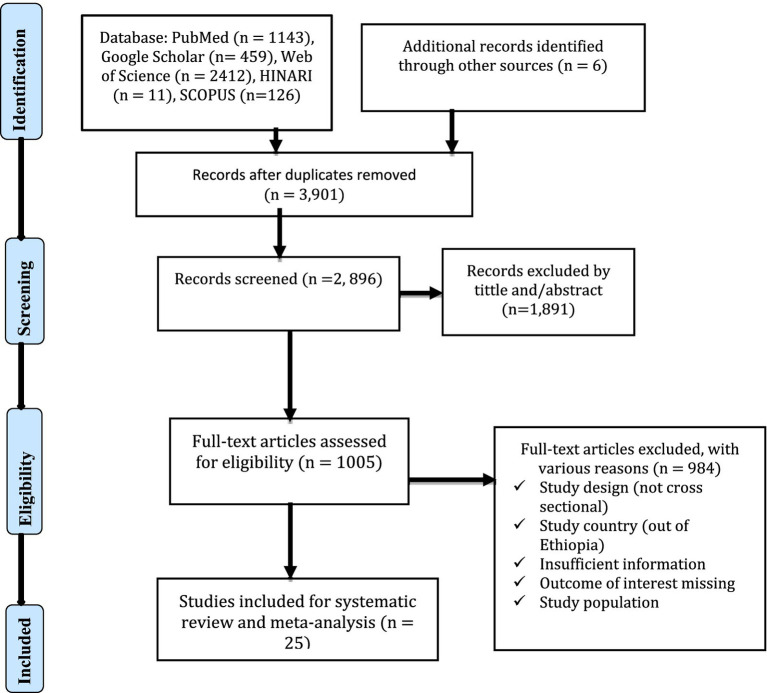
PRISMA flow chart for the included studies.

A comprehensive search was performed in several databases, yielding 4,151 articles, along with 6 additional records identified from other sources (Figure 1). After removing duplicates, 3,901 records remained. Of these, 2,896 records were screened, and 1,891 were excluded based on their title and/or abstract. A total of 1,005 articles were assessed for full-text eligibility, with 984 excluded for various reasons. In the end, twenty five studies (*n* = 25) were included in the meta-analysis.

### Overview of the included articles

The characteristics of studies on *Streptococcus* spp. isolates are detailed as follows. The study subjects were lactating dairy cattle. A total of 25 articles were analyzed for the proportion of *Streptococcus* spp. associated with mastitis, and 54 articles were reviewed on the basis of species isolates. We identified the following isolates: *Str. agalactiae* (*n* = 18; 33%), *Str. dysgalactiae* (*n* = 13; 24%), *Str. uberis* (*n* = 11; 20%), *Str. faecalis* (*n* = 4; 7%), and unidentified *Streptococcus* spp. (*n* = 8; 14.8%). The studies included were conducted in various regions of Ethiopia between 2008 and 2024, predominantly in the southern (Oromia) and central regions. Diagnostic methods included CMT and bacterial culture, following procedures described by (52). The regional distributions of studies were as follows: Oromia (11 studies, 44%), Addis Ababa (7 studies, 27%), SNNPR (2 studies, 8%), Amhara (4 studies, 16%), and Tigray (2 studies, 8%). The minimum sample size was 79 cattle, and the maximum sample size was 1,019 (53). In this review, 7,073 dairy cows were evaluated. The prevalence of bovine mastitis associated with *Streptococcus* infection ranges from 1 to 26% (24, 54), with *Str. uberis* and *Str. agalactiae* being the most prevalent. Of the 25 studies, 18 focused on subclinical mastitis, and 15 focused on clinical mastitis. Three studies (55–57) addressed only subclinical mastitis. The detailed characteristics of the included studies are presented in Table 1.

**Table 1:** Characteristics of the studies included in the meta-analysis (*n* = 25).

First author	Study year	Region	Study design ST	Sample taken	*Str.spp*	TAE	TBI	No. *Str*	Proportion
Dereje et al. (57)	2014–2015	AA	CS, PS	Milk	*Str. agalactiae*	186	97	5	0.052
Dereje et al. (57)	2014–2015	AA	CS, PS	Milk	*Str. dysgalactiae*	186	97	5	0.052
Dereje et al. (57)	2014–2015	AA	CS, PS	Milk	*Str. uberis*	186	97	12	0.124
Etifu and Tilahun (21)	2011–2012	Oromia	CS, SR	Milk	*Str. Sppp*	111	138	8	0.058
Zenebe et al. (89)	2011–2012	Tigray	CS. SR	Milk	*Str. agalactiae*	322	698	142	0.203
Moges et al. (22)	2009–2010	Amhara	CS. SR	Milk	*Str. agalactiae*	322	164	26	0.159
Moges et al. (22)	2009–2010	Amhara	CS. SR	Milk	*Str. dysgalactiae*	322	164	23	0.140
Moges et al. (22)	2009–2010	Amhara	CS. SR	Milk	*Str. uberis*	322	164	11	0.067
Kumbe et al. (59)	2017–2018	Oromia	CS SR	Milk	*Str. Sppp*	330	155	33	0.213
Ararsa et al. (74)	2009–2010	AA	CS. SR	Milk	*Str. agalactiae*	90	180	22	0.122
Ararsa et al. (74)	2009–2010	AA	CS. SR	Milk	*Str. dysgalactiae*	90	180	13	0.072
Ararsa et al. (74)	2009–2010	AA	CS. SR	Milk	*Str. uberis*	90	180	5	0.028
Ararsa et al. (74)	2009–2010	AA	CS. SR	Milk	*Str. faecalis*	90	180	5	0.028
Boggale et al. (53)	2009–2010	Oromia	CS. SR	Milk	*Str. agalactiae*	1,019	1,493	192	0.129
Adane et al. (90)	2010–2011	Oromia	CS, SR	Milk	*Str.spp*	460	641	160	0.250
Tegegne et al. (91)	2015–2016	Amhara	CS SR	Milk	*Str. agalactiae*	303	187	27	0.144
Tegegne et al. (91)	2015–2016	Amhara	CS, SR	Milk	*Str. dysgalactiae*	303	187	11	0.059
Fesseha et al. (92)	2018–2019	Oromia	CS. SR	Milk	*Str.spp*	283	144	16	0.111
Getahun et al. (38)	2007	Oromia	CS, SR	Milk	*Str. agalactiae*	500	195	28	0.144
Getahun et al. (38)	2007	Oromia	CS,SR	Milk	*Str. dysgalactiae*	500	195	6	0.031
Getahun et al. (38)	2007	Oromia	CS SR	Milk	*Str. uberis*	500	195	20	0.103
Girma et al. (39)	2010–2011	Oromia	CS, SR	Milk	*Str. agalactiae*	384	121	24	0.198
Girma et al. (39)	2010–2011	Oromia	CS,SR	Milk	*Str. agalactiae*	384	121	7	0.058
Girma et al. (39)	2010–2011	Oromia	CS, SR	Milk	*Str. uberis*	384	121	7	0.058
Megersa et al. (54)	2009–2010	Sidama	CS, SR	Milk	*Str. agalactiae*	245	200	53	0.265
Mekonnen and Tesfaye (93)	2009	Oromia	CS, SR	Milk	*Str. agalactiae*	206	95	11	0.116
Mekonnen and Tesfaye (93)	2009	Oromia	CS, SR	Milk	*Str. dysgalactiae*	206	95	6	0.063
Mekonnen and Tesfaye (93)	2009	Oromia	CS, SR	Milk	*Str. uberis*	206	95	3	0.032
Mekonnen and Tesfaye (93)	2009	Oromia	CS, SR	Milk	*Str. faecalis*	206	95	10	0.105
Mekibib et al. (94)	2008–2009	AA	CS, SR	Milk	*Str.*spp	107	153	11	0.072
Wubshet et al. (95)	2012–2013	AA	CS.SR	Milk	*Str. agalactiae*	28	72	10	0.139
Wubshet et al. (95)	2012–2013	SNNPR	CS.SR	Milk	*Str. dysgalactiae*	28	72	4	0.056
Wubshet et al. (95)	2012–2013	SNNPR	C.SR	Milk	*Str. uberis*	28	72	5	0.069
Yohannes and Alemu (96)	2017–2018	SNNPR	CS.SR	Milk	*Str. agalactiae*	245	51	9	0.176
Yohannes and Alemu (96)	2017–2018	SNNPR	CS.SR	Milk	*Str. dysgalactiae*	245	51	4	0.078
Tefera et a l. (97)	2019–2021	AA	CS.PS	Milk	*Str.spp*	203	72	12	0.167
Bitew et al. (98)	2009–2010	Amhara.Bdr	CS.Srsm	Milk	*Str. agalactiae*	302	79	7	0.089
Bitew et al. (98)	2009–2010	Amhara	CS.Srsm	Milk	*Str. dysgalactiae*	302	79	4	0.051
Bitew et al. (98)	2009–2010	Amhara	CS.Srsm	Milk	*Str. uberis*	302	79	2	0.025
Haftu et al. (99)	2009–2010	Tigray	CS.SR	Milk	*Str. agalactiae*	305	128	9	0.070
Haftu et al. (99)	2009–2010	Tigray	CS.SR	Milk	*Str. dysgalactiae*	305	128	4	0.031
Zeryehun and Abera (100)	2015–2016	Oromia	CS.SR	Milk	*Str. agalactiae*	384	187	32	0.171
Zeryehun and Abera (100)	2015–2016	Oromia	CS.SR	Milk	*Str. dysgalactiae*	384	187	12	0.064
Zeryehun and Abera (100)	2015–2016	Oromia	CS.SR	Milk	*Str. uberis*	384	187	7	0.037
Melse et al. (101)	2010–2011	Oromia	CS.SR	Milk	*Str.spp*	217	61	10	0.164
Redeat et al. (24)	2019–2021	AA	CS.PS	Milk	*Str. agalactiae*	203	86	8	0.093
Redeat et al. (24)	2019–2021	AA	CS.PS	Milk	*Str. dysgalactiae*	203	86	2	0.023
Redeat et al. (24)	2019–2021	AA	CS.PS	Milk	*Str. uberis*	203	86	1	0.012
Birhanu et al. (55)	2015–2016	AA	CS.SR	Milk	*Str.*spp.*, unidentifed*	262	153	43	0.281
Megersa et al. (54)	2009–2010	Oromia	CS.SR	Milk	*Str. agalactiae*	245	200	53	0.265
Yusuf and Husen (56)	2021	Oromia	CS.SR	Milk	*Str. agalactiae*	56	112	8	0.071
Yusuf and Husen (56)	2021	Oromia	CS,SR	Milk	*Str. uberis*	56	112	3	0.027
Yusuf and Husen (56)	2021	Oromia	CS.SR	Milk	*Strp. faecalis*	56	112	3	0.027

AA, Addis Abeba; CS, cross-sectional; ST, sampling technique; CMT, California mastitis test; SR, simple random sampling; Srsm, systematic random sampling; BC, bacterial culture; TAE, total animal examination; TBI, total bacterial isolation; No. Str, number of *Streptococcus* isolates; PS, purposive sampling.

### Quality assessment results

In this review, a spectrum of studies was evaluated with respect to their quality, which ranged from low to medium proportion. None of the 25 quantitative studies met the criteria set by the AXIS tool, which encompasses details pertaining to risk factors and outcome variables. A majority of the articles, specifically 22 out of 25 (88%), utilized the simple random method procedure outlined by (58). Moreover, 20 studies (80%) successfully obtained a sample frame from a suitable population that closely resembled the target or reference population being investigated. Among the total number of studies, 17 (approximately 68%) fulfilled the requirements for six out of the 20 questions, namely, aims/objectives, definition of target/reference population, internal consistency of results, authors’ justification of the results, sample size justification, analysis of appropriate techniques in the methods and conflicts of interest, and description of the statistical methods used.

### Meta-analysis, heterogeneity testing, and bias assessment

**Figure 2:**
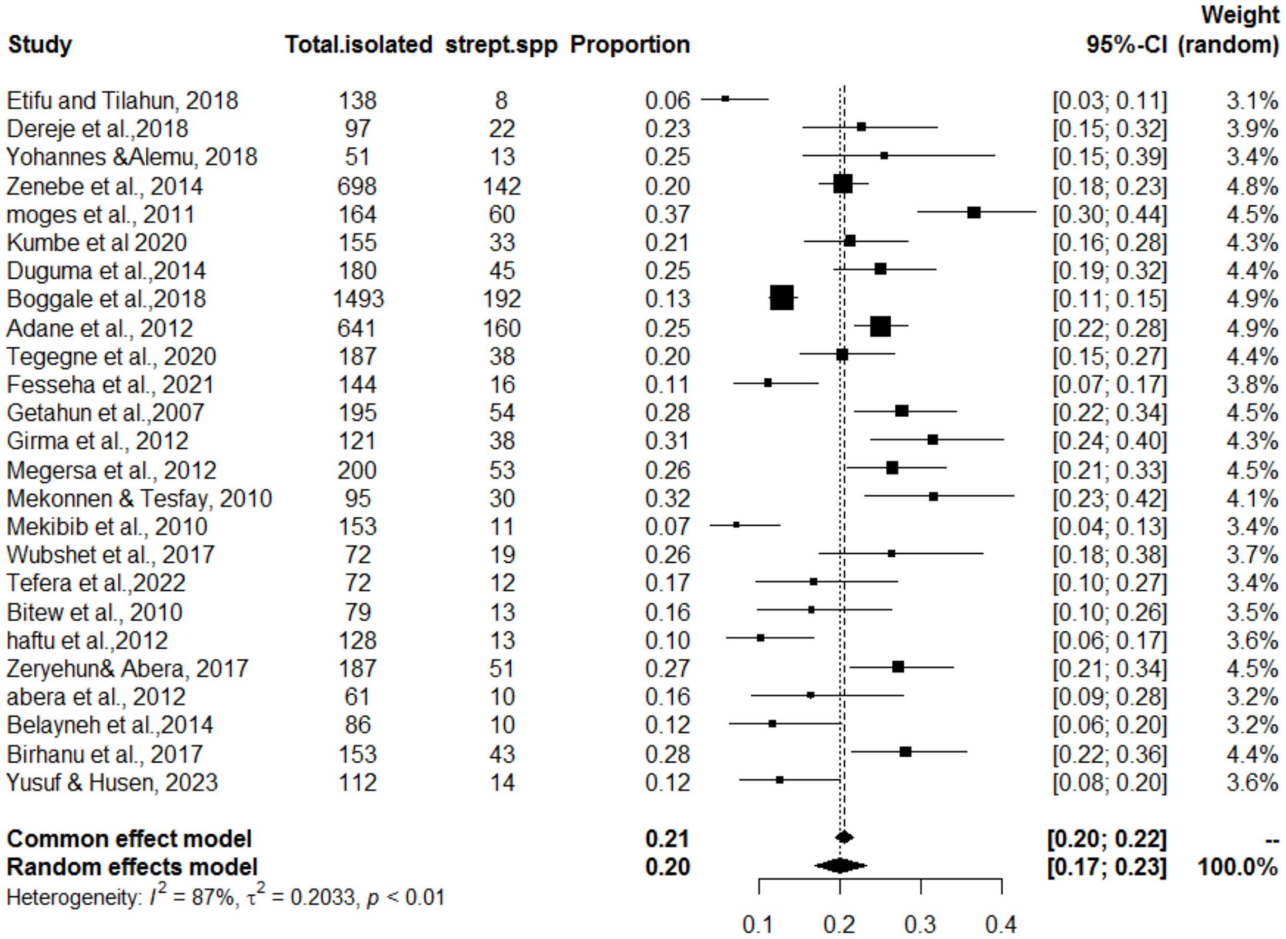
Forest plot for the proportion of *Streptococcus* spp. isolates in dairy cows in Ethiopia. As this figure showed Strep. spp. stands the isolation of *Streptococcus* species isolates.

**Figure 3:**
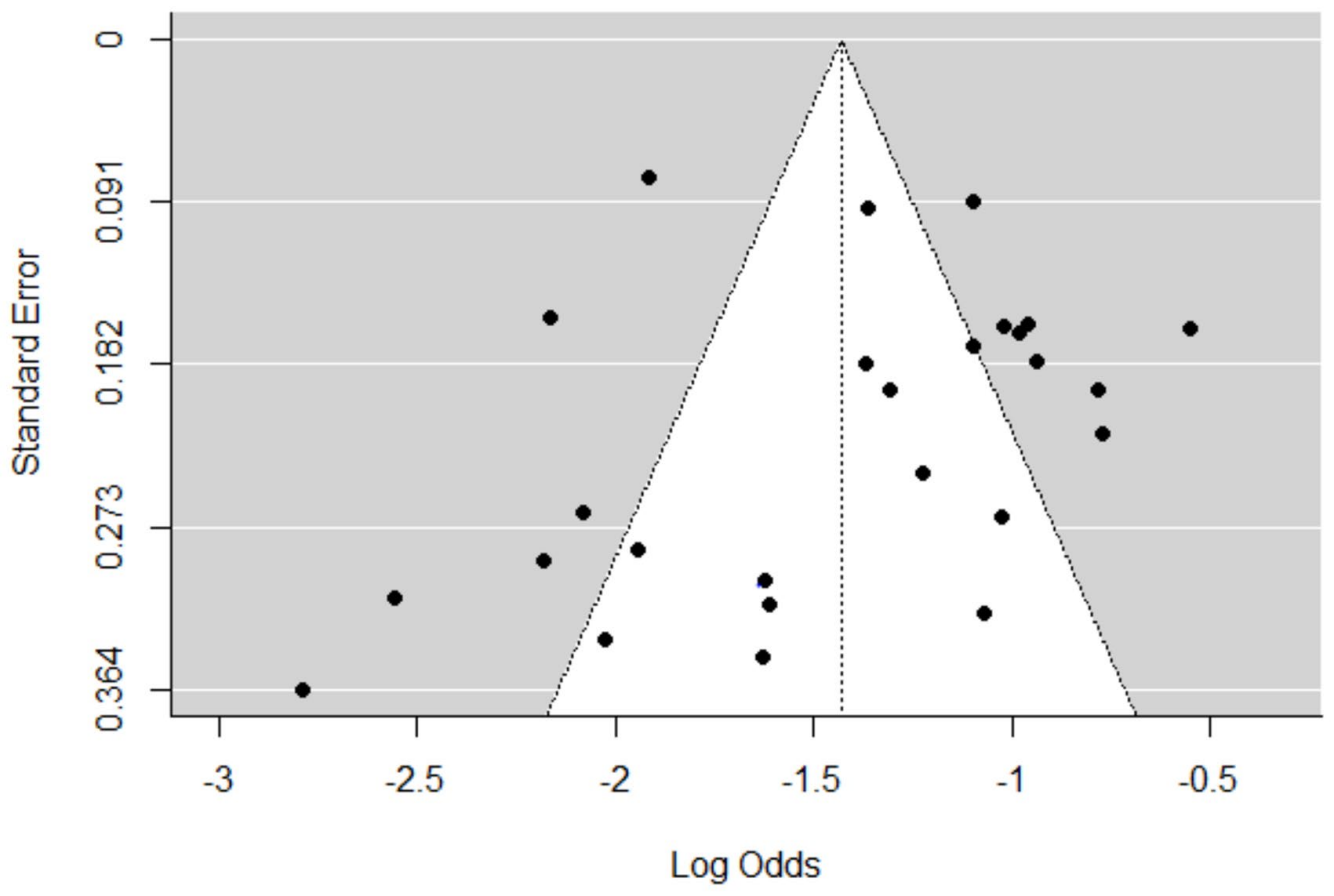
Funnel plots of standard error by log odds of the proportion of *Streptococcus* spp. isolates.

The meta-analysis included 25 articles investigating *Streptococcus* species associated with mastitis. Importantly, some articles were referenced multiple times because of their relevance in similar years but involved investigations of different bacterial strains. The studies included in this analysis showed a substantial level of heterogeneity (I^2^ = 87%, τ^2^ = 0.203; *p* < 0.01). The estimated pooled proportion of *Streptococcus* species associated with mastitis among all bacterial isolates was 20% (95% CI: 17–23%; Figure 2). The variability between studies was statistically significant (Q = 386.5, DF = 25, *p* < 0.001). The funnel plots (Figure 3) and Egger’s regression asymmetry did not suggest the presence of publication bias (*p* > 0.05).

### Subgroup analysis

Because of high degree of heterogeneity, sub analyses were conducted on the basis of the study location or region, study year, degree of mastitis, and type of *Streptococcus* spp., as shown in Table 2 and Supplementary Figures 1–4. Significant heterogeneity between studies was found in the sub analysis by region-wise (*p* < 0.001). The subgroup analysis of *Streptococcus* bacteria associated to bovine mastitis by region revealed that SNNPR had the largest pooled proportion of mastitis-associated *Streptococcus* isolates (26%), followed by Amhara (24%), Oromia (19%), AA (18%), and Tigray (15%). However, Oromia region had the highest heterogeneity (I^2^ = 91%; *p* < 0.01) across studies. A sub analysis was conducted regarding the study year (studies grouped into before 2013 and studies after 2013). The magnitude of heterogeneity were I^2^ = 83% and I^2^ = 86%, respectively. The highest sub pooled proportion of *Streptococcus* isolates associated to mastitis (20%) occurred prior to 2013. The subgroup difference test results (Q = 0.18; DF = 1; *p* = 0.743) indicated the absence of a statistically significant group effect.

**Table 2:** Pooled estimates of *Streptococcus* spp., stratified by subgroups.

Moderators	K	Category	N	Case	ES (95%CI)(RE)	Heterogeneity			Test for subgroup differences (RE)	
						I^2^ (%)	τ^2^	p value	Q	*p* value
Pooled.ES	25	Overall	5,662	1,100	0.20 (0.17;0.23)	87	0.20	<0.01	207.9	<0.0001
Species-wise	13	*Str. dysagalcte*	1,642	101	0.06 (0.046; 0.079)	49	0.12	0.02	46.79	<0.0001
	16	*Str. agalactie*	4,791	712	0.13 (0.109; 0.161)	79	0.14	<0.001		
	11	*Str. ubreis*	1,388	76	0.055 (0.04; 0.080)	60	0.25	<0.01		
	3	*Str. Faecalis*	387	18	0.046 (0.018; 0.116)	78	0.57	0.01		
	8	Un-identified	1,517	293	0.15 (0.103; 0.224)	87	0.37	<0.01		
Level mastitis	18	Clinical	1823	625	0.24 (0.116; 0.306)	95.9	162	<0.001	0.45	0.5023
	18	Subclinical	3,839	735	0.18 (0.101; 0.231)	96.8	1.08	<0.001		
Year-wise	12	Post-2016	2, 809	463	0.19 (0.150; 0.270)	83	0.14	0.00	0.11	0.74
	13	Pre-2016	2, 853	637	0.20 (0.029; 0.192)	86	0.39	<0.01		
Region-wise	11	Oromia	3,342	606	0.19 (0.1419; 0.25)	91	1.25	<0.01	5.56	0.021
	3	Amhara	430	111	0.24 (0.143; 0.375)	87	1.12	<0.01		
	2	Tigray	826	155	0.15 (0.075; 0.283)	86	1.35	<0.01		
	7	AA	813	162	0.19 (0.131; 0.264)	79	0.27	<0.01		
	2	SNNPR	251	66	0.26 (0.212; 0.321)	0	0	0.88		

K, Number of included studies; N, Total number of isolates; Case, *Streptococcus* spp. isolates; SNNPR, South Nations, Nationalities and peoples Region; AA, Addis Ababa; ES, Effect size; RE, random effect.

A sub-analysis on the basis of the degree of mastitis also revealed that, with study weights of 40 and 59.5%, the percentage of *Streptococcus* isolates at the species level was greater in clinical mastitis at 24% (95% CI; 16–34%) than in subclinical bovine mastitis at 18% (95% CI; 14–24%). Both the clinical and subclinical mastitis categories experienced significant study variability across studies (I^2^ = 89%; *p* < 0.01) and (I^2^ = 87%; p < 0.01), respectively. Five groups were formed from the sub-analysis of the included studies on the basis of the types of *Streptococcus* species: *Str. agalactiae* (*n* = 18), *Str. dysgalactiae* (*n* = 13), *Str. uberis* (*n* = 11), *Str. Faecalis* (*n* = 4) and unknown *Streptococcus* spp. (*n* = 8). Several publications did not identify the species level (21, 55, 59, 60). In the sub-analysis of the proportion of *Streptococcus* associated with mastitis by species type, there were notable differences. According to the subgroup analysis proportion shown in Supplementary Figure 4, the pooled proportion of unidentified spp. was the next highest at 15% (95% CI: 10–22%) and (I^2^ = 87%: τ^2^ = 0.370; *p* < 0.01) for *Str. agalactie*. 13% (95% CI: 11–16%) and (I^2^ = 79%) (τ^2^ = 0.146; *p* < 0.01). In the present meta-analysis, the proportion of *Str. dysgalactiae* was found to be 6% (95% CI: 5–8%) with low heterogeneity (I^2^ = 49%: τ^2^ = 0.126; *p* = 0.02).

In all included studies, the Kirby-Baur disc diffusion method was used as an antimicrobial sensitivity test. The present studies on antimicrobial resistance in *Streptococcus* for the treatment of mastitis in cattle are depicted in Table 3. Only studies accurately proportionally identifying *Streptococcus* species to the species level were included in the meta-analysis. This approach helped to exclude studies that may have misclassified or grouped different species, which could have had varying resistance profiles.

**Table 3:** Summary of included studies on antimicrobial resistance.

Author	Antimicrobial	Group	*Str*. spp	Total	No. resistance	Proportion
Boggale et al. (53)	Amoxicillin	Beta lactam	*Str. agalactiae*	192	41	0.214
Boggale et al. (53)	Amoxicillin	Beta lactam	*Str. faecalis*	38	11	0.289
Girma et al. (39)	Amoxicillin	Beta lactam	*Str. agalactiae*	16	1	0.063
Girma et al. (39)	Amoxicillin	Beta lactam	*Str. dysgalactiae*	7	2	0.286
Girma et al. (39)	Amoxicillin	Beta lactam	*Str. uberis*	5	1	0.200
Moges et al. (22)	Ampicillin	Beta lactam	*Str. agalactiae*	20	8	0.400
Moges et al. (22)	Ampicillin	Beta lactam	*Str. dysgalactiae*	15	3	0.2000
Moges et al. (22)	Ampicillin	Beta lactam	*Str. uberis*	7	3	0.429
Etifu et al. (102)	Ampicillin	Beta lactam	*Unidentified Str*	8	2	0.2500
Boggale et al. (53)	Ampicillin	Beta lactam	*Str. agalactiae*	192	52	0.271
Boggale et al. (53)	Ampicillin	Beta lactam	*Str. faecalis*	38	18	0.474
Girma et al. (39)	Ampicillin	Beta lactam	*Str. agalactiae*	16	8	0.500
Girma et al. (39)	Ampicillin	Beta lactam	*Str. dysgalactiae*	7	4	0.571
Girma et al. (39)	Ampicillin	Beta lactam	*Str. uberis*	5	2	0.4
Getahun et al. (38)	Ampicillin	Beta lactam	*Str. agalactiae*	13	2	0.154
Getahun et al. (38)	Ampicillin	Beta lactam	*Str. dysgalactiae*	3	0	0.000
Getahun et a (38).	Ampicillin	Beta lactam	*Str. uberis*	19	7	0.368
Boggale et al. (53)	Cloxacillin	Beta lactam	*Str. agalactiae*	192	69	0.359
Boggale et al. (53)	Cloxacillin	Beta lactam	*Str. faecalis*	38	23	0.605
Girma et al. (39)	Cloxacillin	Beta lactam	*Str. agalactiae*	16	2	0.125
Girma et al. (39)	Cloxacillin	Beta lactam	*Str. dysgalactiae*	7	1	0.143
Girma et al. (39)	Cloxacillin	Beta lactam	*Str. uberis*	5	1	0.200
Dereje et al. (57)	Cotrimoxazole	Sulphonamide	*Str. agalactiae*	5	1	0.200
Etifu et al. (102)	Cotrimoxazole	Sulphonamide	*str. spp*	8	1	0.125
Dereje et al. (57)	Cotrimoxazole	Sulphonamide	*Str. dysgalactiae*	5	1	0.200
Dereje et al. (57)	Cotrimoxazole	Sulphonamide	*Str. uberis*	12	4	0.333
Dereje et al. (57)	Erythromycin	Aminoglycosides	*Str. agalactiae*	5	2	0.400
Moges et al. (22)	Erythromycin	Aminoglycosides	*Str. agalactiae*	20	4	0.200
Moges et al. (22)	Erythromycin	Aminoglycosides	*Str. dysgalactiae*	15	3	0.200
Moges et al. (22)	Erythromycin	Aminoglycosides	*Str. uberis*	7	4	0.571
Etifu et al. (102)	Erythromycin	Aminoglycosides	*Unidentified/strep*	8	0	0.000
Dereje et al. (57)	Erythromycin	Aminoglycosides	*Str. dysgalactiae*	5	0	0.00
Dereje et al. (57)	Erythromycin	Aminoglycosides	*Str. uberis*	12	0	0.00
Getahun et al. (38)	Erythromycin	Aminoglycosides	*Str. agalactiae*	13	1	0.077
Getahun et al. (38)	Erythromycin	Aminoglycosides	*Str. dysgalactiae*	3	1	0.333
Getahun et al. (38)	Erythromycin	Aminoglycosides	*Str. uberis*	19	0	0.00
Dereje et al. (57)	Gentamycin	Aminoglycosides	*Str. agalactiae*	5	0	0.00
Etifu et al. (102)	Gentamycin	Aminoglycosides	*Unidentified strep*	8	0	0.00
Boggale et al. (53)	Gentamycin	Aminoglycosides	*Str. agalactiae*	192	30	0.156
Boggale et al. (53)	Gentamycin	Aminoglycosides	*Str. faecalis*	38	7	0.184
Dereje et al. (57)	Gentamycin	Aminoglycosides	*Str. dysgalactiae*	5	0	0.00
Dereje et al. (57)	Gentamycin	Aminoglycosides	*Str. uberis*	12	2	0.167
Girma et al. (39)	Gentamycin	Aminoglycosides	*Str. agalactiae*	16	2	0.125
Girma et al. (39)	Gentamycin	Aminoglycosides	*Str. dysgalactiae*	7	2	0.286
Girma et al. (39)	Gentamycin	Aminoglycosides	*Str. uberis*	5	0	0.00
Moges et al. (22)	Oxytetracycline	Tetracycline	*Str. agalactiae*	20	5	0.25
Moges et al. (22)	Oxytetracycline	Tetracycline	*Str. dysgalactiae*	15	11	0.733
Moges et al. (22)	Oxytetracycline	Tetracycline	*Str. uberis*	7	0	0.00
Boggale et al. (53)	Oxytetracycline	Tetracycline	*Str. agalactiae*	192	49	0.255
Boggale et al. (53)	Oxytetracycline	Tetracycline	*S. faecalis*	38	15	0.395
Girma et al. (39)	Oxytetracycline	Tetracycline	*Str. agalactiae*	16	7	0.438
Girma et al. (39)	Oxytetracycline	Tetracycline	*Str. dysgalactiae*	7	4	0.571
Girma et al. (39)	Oxytetracycline	Tetracycline	*Str. uberis*	5	3	0.600
Getahun et al. (38)	Oxytetracycline	Tetracycline	*Str. agalactiae*	13	4	0.308
Getahun et al. (38)	Oxytetracycline	Tetracycline	*Str. dysgalactiae*	3	0	0.000
Getahun et al. (38)	Oxytetracycline	Tetracycline	*Str. uberis*	19	2	0.105
Dereje et al. (57)	Pencillin	Beta lactam	*Str. agalactiae*	5	1	0.200
Boggale et al. (53)	Pencillin	Beta lactam	*Str. agalactiae*	192	89	0.464
Boggale et al. (53)	Pencillin	Beta lactam	*Str. faecalis*	38	30	0.789
Dereje et al. (57)	Pencillin	Beta lactam	*Str. dysgalactiae*	5	2	0.400
Dereje et al. (57)	Pencillin	Beta lactam	*Str. uberis*	12	4	0.333
Girma et al. (39)	Pencillin	Beta lactam	*Str. agalactiae*	16	12	0.750
Girma et al. (39)	Pencillin	Beta lactam	*Str. dysgalactiae*	7	6	0.857
Girma et al. (39)	Pencillin	Beta lactam	*Str. uberis*	5	4	0.800
Getahun et al. (38)	Pencillin	Beta lactam	*Str. agalactiae*	13	2	0.154
Getahun et al. (38)	Pencillin	Beta lactam	*Str. dysgalactiae*	3	2	0.667
Getahun et al. (38)	Pencillin	Beta lactam	*Str. uberis*	19	7	0.368
Moges et al. (22)	Sulphonamide	Sulphonamide	*Str. agalactiae*	20	0	0.000
Moges et al. (22)	Sulphonamide	Sulphonamide	*Str. dysgalactiae*	15	5	0.33
Moges et al. (22)	Sulphonamide	Sulphonamide	*Str. uberis*	7	0	0.00
Getahun et al. (38)	Sulphonamide	Sulphonamide	*Str. agalactiae*	13	2	0.154
Getahun et al. (38)	Sulphonamide	Sulphonamide	*Str. dysgalactiae*	3	0	0.00
Getahun et al. (38)	Sulphonamide	Sulphonamide	*Str. uberis*	19	6	0.316
Dereje et al. (57)	Tetracycline	Tetracycline	*Str. agalactiae*	5	1	0.200
Moges et al. (22)	Tetracycline	Tetracycline	*Str. agalactiae*	20	8	0.400
Moges et al. (22)	Tetracycline	Tetracycline	*Str. dysgalactiae*	15	9	0.600
Moges et al. (22)	Tetracycline	Tetracycline	*Str. uberis*	7	3	0.429
Etifu et al. (102)	Tetracycline	Tetracycline	*Unidentified*	8	1	0.125
Dereje et al. (57)	Tetracycline	Tetracycline	*Str. dysgalactiae*	5	0	0.00
Dereje et al. (57)	Tetracycline	Tetracycline	*Str. uberis*	12	4	0.333

### Pooled estimates of antimicrobial resistance

The highest prevalence of resistant *Streptococcus* was *against* penicillin (*pool* estimate = 52, 95% CI = 38–67%), followed by streptomycin, tetracycline, and ampicillin (*pool* estimates = 42, 38, and 35%, respectively). Gentamycin and erythromycin presented the lowest overall prevalence of resistance (pool estimates = 16 and 19%, respectively) (Table 3). In general, the *I^2^* values were highest for the streptomycin antimicrobials tested (*I^2^* = 80%). The *I^2^* of amoxicillin, Co-trimazole and gentamycin were equal to zero since they were used in any of the included studies; therefore, there was no variability (Table 4).

**Table 4:** Overall pooled estimate of the prevalence of *Streptococcus* AMR to specific antimicrobial agents.

Type of antimicrobial	Total isolates	No. resistance isolates	Pooled resistance (95%)	Heterogeneity%(*I^2^*)	*p* value	Tau^2^
Amoxacillin	258	56	22 (18–28%)	0	0.52	0.000
Ampicillin	343	102	35 (28–43%)	26	0.19	0.077
Cloxacillin	258	96	33 (18–52%)	71	<0.01	0.4731
Cotrimazole	30	7	22 (12–44%)	0	0.75	0.000
Erythromycin	107	15	19 (10–33%)	31	0.16	0.3934
Gentamycin	288	43	16 (12–20%)	0	0.96	0.000
Pencillin	315	159	52 (38–67%)	66	<0.01	0.5339
Streptomycin	72	26	42 (26–61)	80	<0.01	1.0550
Tetracycline	335	111	38 (25–52%)	17	0.30	0.1060
Oxytetracycline	335	100	35 (24–47%)	59	<0.01	0.3429
Sulphonamides	77	13	21 (11–36%)	22	0.27	0.1883

## Discussion

This meta-analysis, comprising twenty-five observational studies, revealed that 20% of bovine mastitis cases in Ethiopia were associated with *Streptococcus* spp. Among the various species studied, the prevalence of *Str. agalactiae* was 13%, followed by *Str. dysgalactiae* at 6% and *Str. uberis* at 5%. *Str. agalactiae* had the highest prevalence, likely because it is a highly contagious obligate pathogen of the bovine mammary gland (61). The overall prevalence (20%) is similar to findings from the United States (20.8–23.3%), Bangladesh (28.75%) (62), and the Netherlands (25%) but lower than reports from China (36.23%) (63), Nigeria (56.7%), Egypt (38.3%), Tanzania (75.5%) (64), Europe (38%), Australia (50%), France (42.11%), and New Zealand (58.66%) (65, 66). This variability may be due to differences in climate, knowledge levels, management systems, cow breeds, laboratory facilities, and housing styles across countries (67). The pooled prevalence of *Str. agalactiae* (13%) and *Str. dysgalactiae* (6%) was lower than that in Bangladesh, where the prevalence of *Str. agalactiae* was 19.86% and that of *Str. dysgalactiae* was 17.81% (68), and in Egypt, where the prevalence of *Str. dysgalactiae* was 23% and that of *Str. agalactiae* was 20.1% (69). These disparities could be due to differences in geographic location, livestock rearing, husbandry, and hygiene practices (70–72). Subgroup analysis by region in Ethiopia revealed the highest prevalence of streptococcal infection in SNNPRS (26%) and the lowest in Tigray (15%). This variation could be due to differences in agroclimatic conditions, sampling methods, farm management practices, and cow-related factors. In terms of mastitis severity, this meta-analysis revealed a greater occurrence of streptococcal infection in clinical mastitis cases (24%) than in subclinical mastitis cases (18% in CMT-positive cows). Our findings show that clinical mastitis caused by *Streptococcus* is more prevalent than subclinical mastitis, contrary to some reports (59, 60, 73), but consistent with others (38, 39, 74).

In recent years, the proportion of streptococcal infection in bovine mastitis has decreased, likely due to increased awareness through scientific training, research, technological advancements, and the implementation of biosecurity measures on farms. Furthermore, studies from China have demonstrated that subclinical mastitis is a significant issue in smallholder dairy farms, with a variety of bacterial pathogens, including *Streptococcus* spp., playing a major role in infections (75). The molecular characterization of antimicrobial-resistant pathogens such as *Staphylococcus haemolyticus* in dairy herds of Northwest China indicates that the dairy environment can act as a reservoir for resistant bacterial strains, further complicating treatment strategies (76).

The second aim of this review was to determine the antimicrobial resistance (AMR) profile of *Streptococcus* spp. in bovine mastitis. The pooled resistance proportion to penicillin was 52%, which was much higher than that reported in Uruguay (28.6%) (77), France (21%) (78), and Argentina (27.6%) (79). The antimicrobials that have been showing the greatest resistance were aminoglycosides, streptomycin, and penicillin, likely due to long-term and repeated use on dairy farms. The resistance proportions for erythromycin and tetracycline were 19 and 38%, respectively, similar to findings in France (20 and 38.5%) (80, 81) but lower than the resistance proportions reported in the USA and Europe, which ranged from 20 to 50% (82, 83). The resistance of *Streptococcu*s spp. to gentamycin (16%) and tetracycline (38%) was greater than the proportions reported by (81) (2.4 and 18%, respectively). The 38% resistance proportion to tetracycline in this study is lower than the proportion reported in Denmark between 2002 and 2004 (84, 75.5, and 84.8%) (84, 85). Recent findings highlight that dairy farms are a potential reservoir for antibiotic-resistant bacteria and virulence genes, particularly among *Escherichia coli* strains that carry resistance to aminoglycosides and beta-lactam antibiotics (86). The One Health approach to AMR suggests that the transmission of resistant bacteria is not confined to animals but extends to the broader ecosystem, including farm workers and the surrounding environment. The rise of multidrug-resistant strains in dairy environments underscores the need for stringent antimicrobial stewardship (87).

The variation in antimicrobial resistance across regions may be attributed to differences in medication practices, with improper use of antimicrobial drugs being a significant contributor to the development of resistance (88). Our findings indicate a high prevalence of both contagious and environmental *Streptococcu*s spp. in bovine mastitis in Ethiopia. Therefore, an extended ten-point mastitis control plan should be implemented, with components tailored specifically for Ethiopia, including increased awareness among farmers and milkers. Additionally, targeted interventions for regions with high infection proportions, research into alternative therapeutic approaches, and the development of new antimicrobials are critical measures that must be undertaken.

### Limitations of the included articles and this systematic review

Most of the articles describe the frequency of the isolates and the percentage of the resistance isolates however no articles tried to identify the resistance genes. Most of the articles used the convenience method of the sample selection so it may lead to selection biased. This systematic review has several limitations, which must be taken into consideration. First, the review is focused exclusively on a single genus, namely, *Streptococcus*. Second, few studies were included in the analysis of antimicrobial resistance. Third, no studies were included in some regions. Finally, the protocol was not registered in the PROSPERO database.

## Conclusion and future perspectives

The present meta-analysis showed the overall pooled proportion of mastitis associated with *Streptococcus* spp. at 20% (95% CI: 17–23%). The highest proportions were found in SNNPR (26%), followed in Amhara (24%), Oromia and Addis Ababa (19%), and Tigray (15%). Clinical mastitis had the highest proportion of streptococcal isolates (24%). *Str. agalactiae* had the highest pooled prevalence at 13%. Resistance was highest against penicillin (52%), followed by streptomycin (42%), tetracycline (38%), and ampicillin (35%). Specifically, *Str. agalactiae* accounted for the highest proportion of bovine mastitis-causing *Streptococcus* spp. infections. This particular species of *Streptococcus* falls under the category of a contagious group of bacterial pathogens, indicating that a notable proportion of contagious *Streptococcus* and the udder of infected cows serve as a significant reservoir. Among the commonly employed antimicrobials, the highest pooled resistance proportion of *Streptococcus* spp. was observed against penicillin. The data presented in this report will facilitate informed decision-making processes aimed at controlling and preventing bovine mastitis within the context of Ethiopia. The findings will benefit stakeholders and policymakers in enhancing the dairy industry. Increased monitoring and reporting of streptococcal mastitis and antimicrobial resistance across Ethiopia will improve the understanding of prevalence and resistance patterns. The implementation of stricter protocols for Antimicrobial use, especially those that reduce the reliance on highly resistant antimicrobials such as penicillin, is essential. Developing targeted interventions for regions with relatively high infection proportions, promoting research into alternative therapies, and innovating new antimicrobials are also critical steps.

## Data Availability

The original contributions presented in the study are included in the article/Supplementary material, further inquiries can be directed to the corresponding author.
