# Supplementary material for: Streptococcal infection and its antimicrobial resistance profile associated with bovine mastitis in Ethiopia: a systematic review and meta-analysis
Source: Front Vet Sci. 2025 Mar 12;12:1503904. doi: 10.3389/fvets.2025.1503904 (PMC11938844; doi:10.3389/fvets.2025.1503904)
Supplement: Supplementary file 1 [file Image_1.pdf]

Supplementary sub group Strep

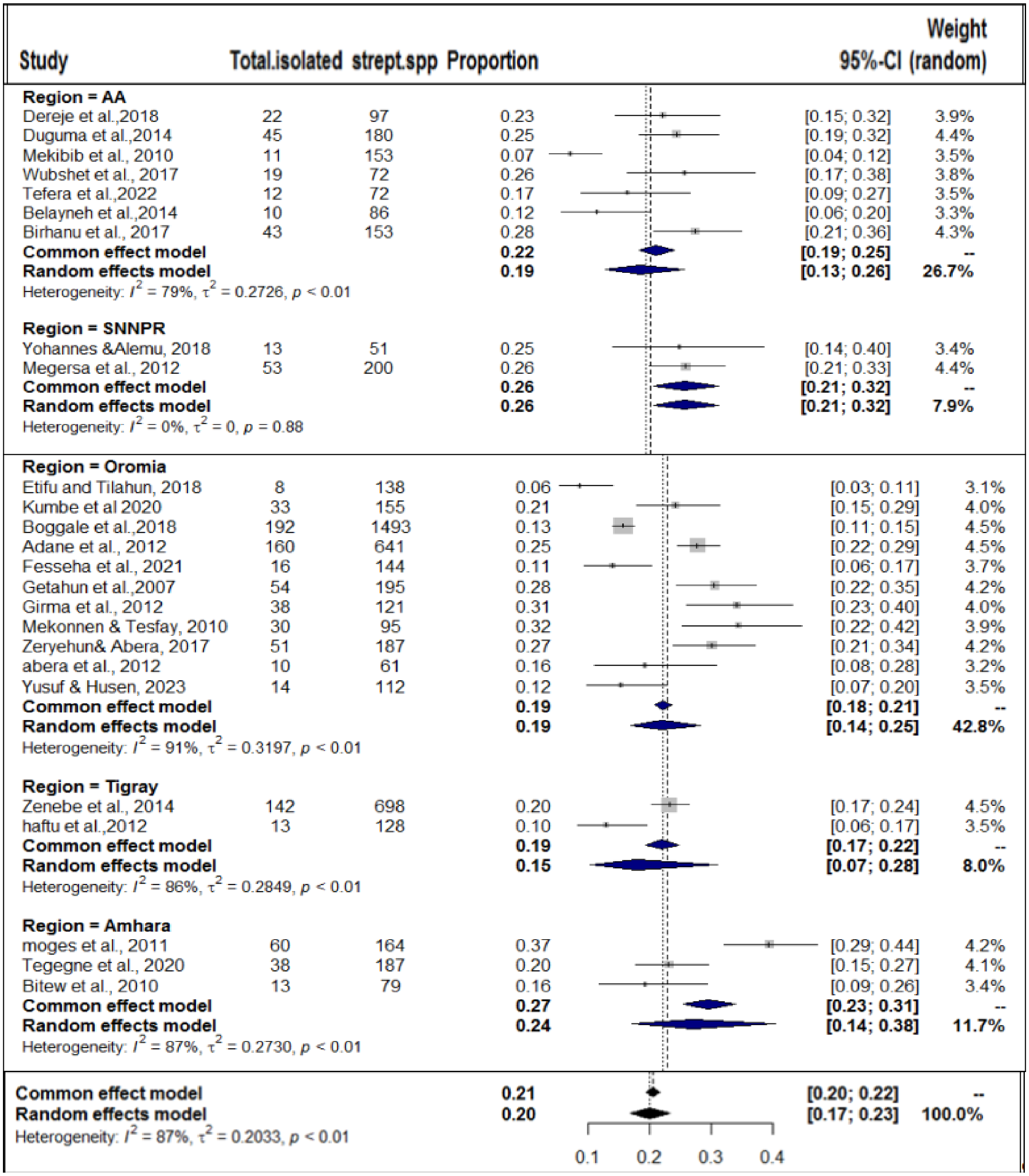

Figure 1 Subgroup analysis of the proportion of mastitis-associated *Sterptococcus* by study region

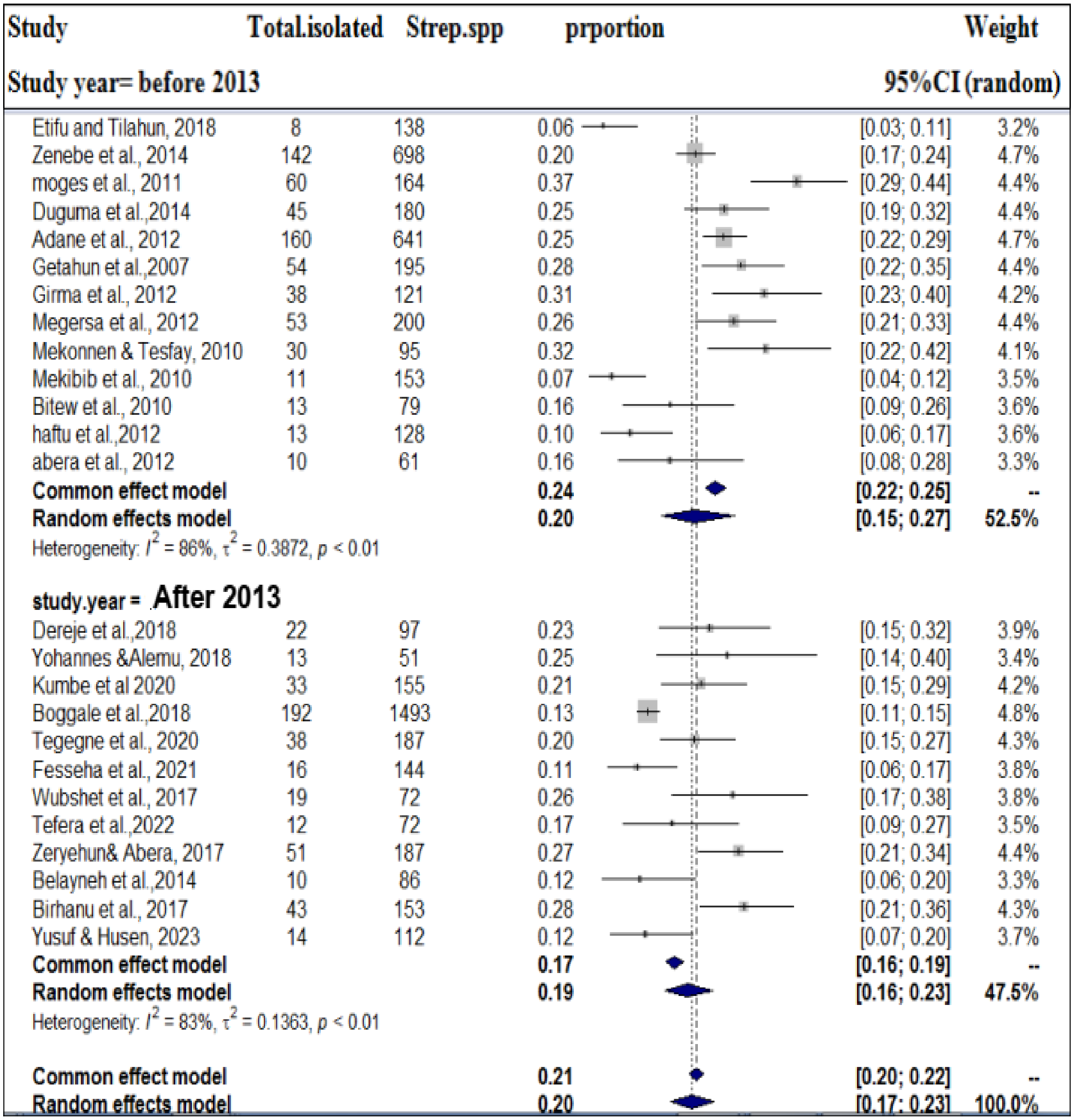

Figure 2 Subgroup analysis of the proportion of mastitis-associated *Sterptococcus* by year of study

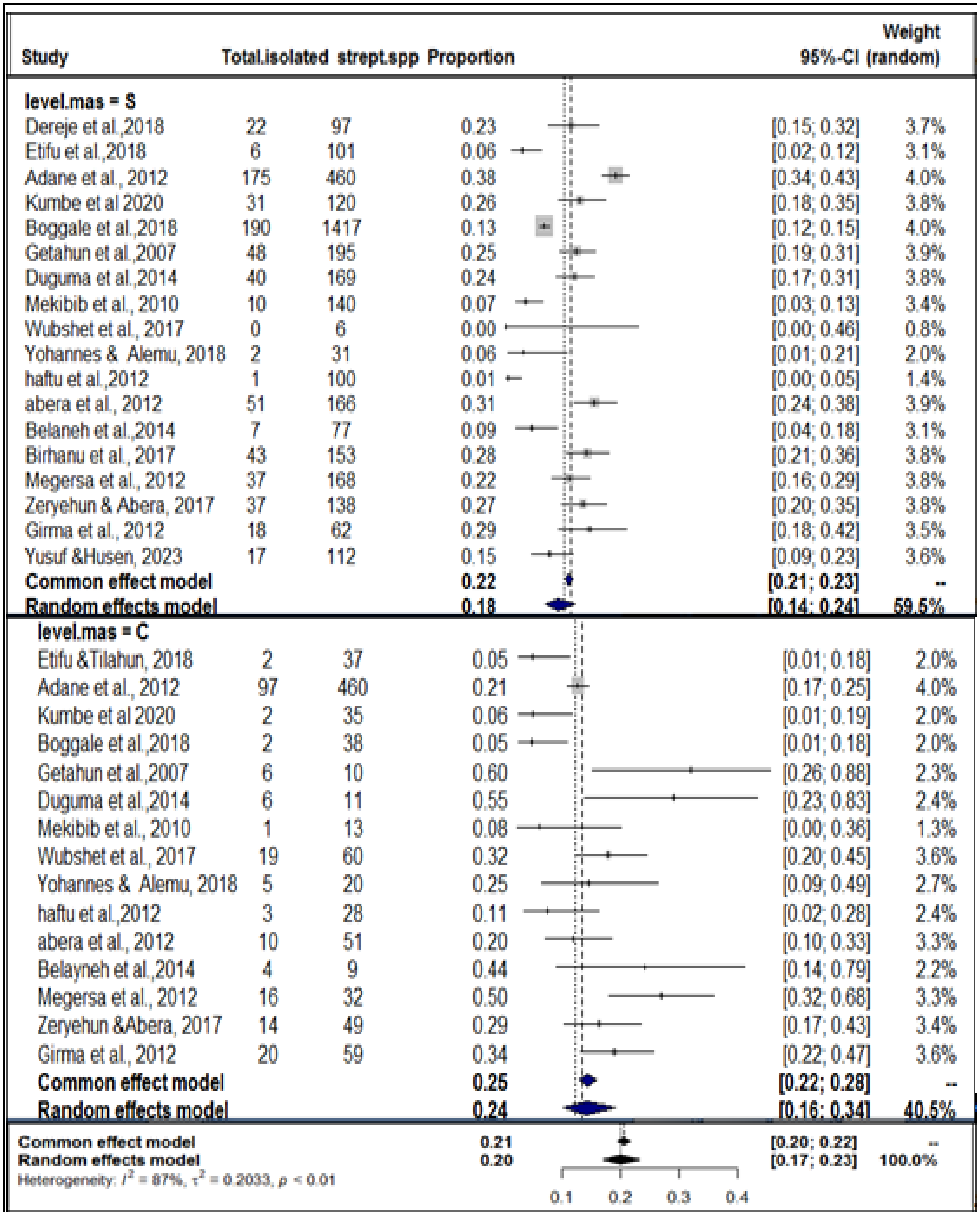
Study Total.isolated strept.spp Proportion 95%-CI Weight (random)
level.mas = S
Dereje et al.,2018 22 97 0.23 [0.15; 0.32] 3.7%
Etifu et al.,2018 6 101 0.06 [0.02; 0.12] 3.1%
Adane et al., 2012 175 460 0.38 [0.34; 0.43] 4.0%
Kumbe et al 2020 31 120 0.26 [0.18; 0.35] 3.8%
Boggale et al.,2018 190 1417 0.13 [0.12; 0.15] 4.0%
Getahun et al.,2007 48 195 0.25 [0.19; 0.31] 3.9%
Duguma et al.,2014 40 169 0.24 [0.17; 0.31] 3.8%
Mekibib et al., 2010 10 140 0.07 [0.03; 0.13] 3.4%
Wubshet et al., 2017 0 6 0.00 [0.00; 0.46] 0.8%
Yohannes & Alemu, 2018 2 31 0.06 [0.01; 0.21] 2.0%
haftu et al.,2012 1 100 0.01 [0.00; 0.05] 1.4%
abera et al., 2012 51 166 0.31 [0.24; 0.38] 3.9%
Belaneh et al.,2014 7 77 0.09 [0.04; 0.18] 3.1%
Birhanu et al., 2017 43 153 0.28 [0.21; 0.36] 3.8%
Megersa et al., 2012 37 168 0.22 [0.16; 0.29] 3.8%
Zeryehun & Abera, 2017 37 138 0.27 [0.20; 0.35] 3.8%
Girma et al., 2012 18 62 0.29 [0.18; 0.42] 3.5%
Yusuf &Husen, 2023 17 112 0.15 [0.09; 0.23] 3.6%
Common effect model 0.22 [0.21; 0.23] --
Random effects model 0.18 [0.14; 0.24] 59.5%
level.mas = C
Etifu &Tilahun, 2018 2 37 0.05 [0.01; 0.18] 2.0%
Adane et al., 2012 97 460 0.21 [0.17; 0.25] 4.0%
Kumbe et al 2020 2 35 0.06 [0.01; 0.19] 2.0%
Boggale et al.,2018 2 38 0.05 [0.01; 0.18] 2.0%
Getahun et al.,2007 6 10 0.60 [0.26; 0.88] 2.3%
Duguma et al.,2014 6 11 0.55 [0.23; 0.83] 2.4%
Mekibib et al., 2010 1 13 0.08 [0.00; 0.36] 1.3%
Wubshet et al., 2017 19 60 0.32 [0.20; 0.45] 3.6%
Yohannes & Alemu, 2018 5 20 0.25 [0.09; 0.49] 2.7%
haftu et al.,2012 3 28 0.11 [0.02; 0.28] 2.4%
abera et al., 2012 10 51 0.20 [0.10; 0.33] 3.3%
Belayneh et al.,2014 4 9 0.44 [0.14; 0.79] 2.2%
Megersa et al., 2012 16 32 0.50 [0.32; 0.68] 3.3%
Zeryehun &Abera, 2017 14 49 0.29 [0.17; 0.43] 3.4%
Girma et al., 2012 20 59 0.34 [0.22; 0.47] 3.6%
Common effect model 0.25 [0.22; 0.28] --
Random effects model 0.24 [0.16; 0.34] 40.5%
Common effect model 0.21 [0.20; 0.22] --
Random effects model 0.20 [0.17; 0.23] 100.0%
Heterogeneity: I^2 = 87%, τ^2 = 0.2033, p < 0.01
0.1 0.2 0.3 0.4

Figure 3 Subgroup analysis of the proportion of mastitis-associated S*treptococcus* strains stratified by mastitis severity

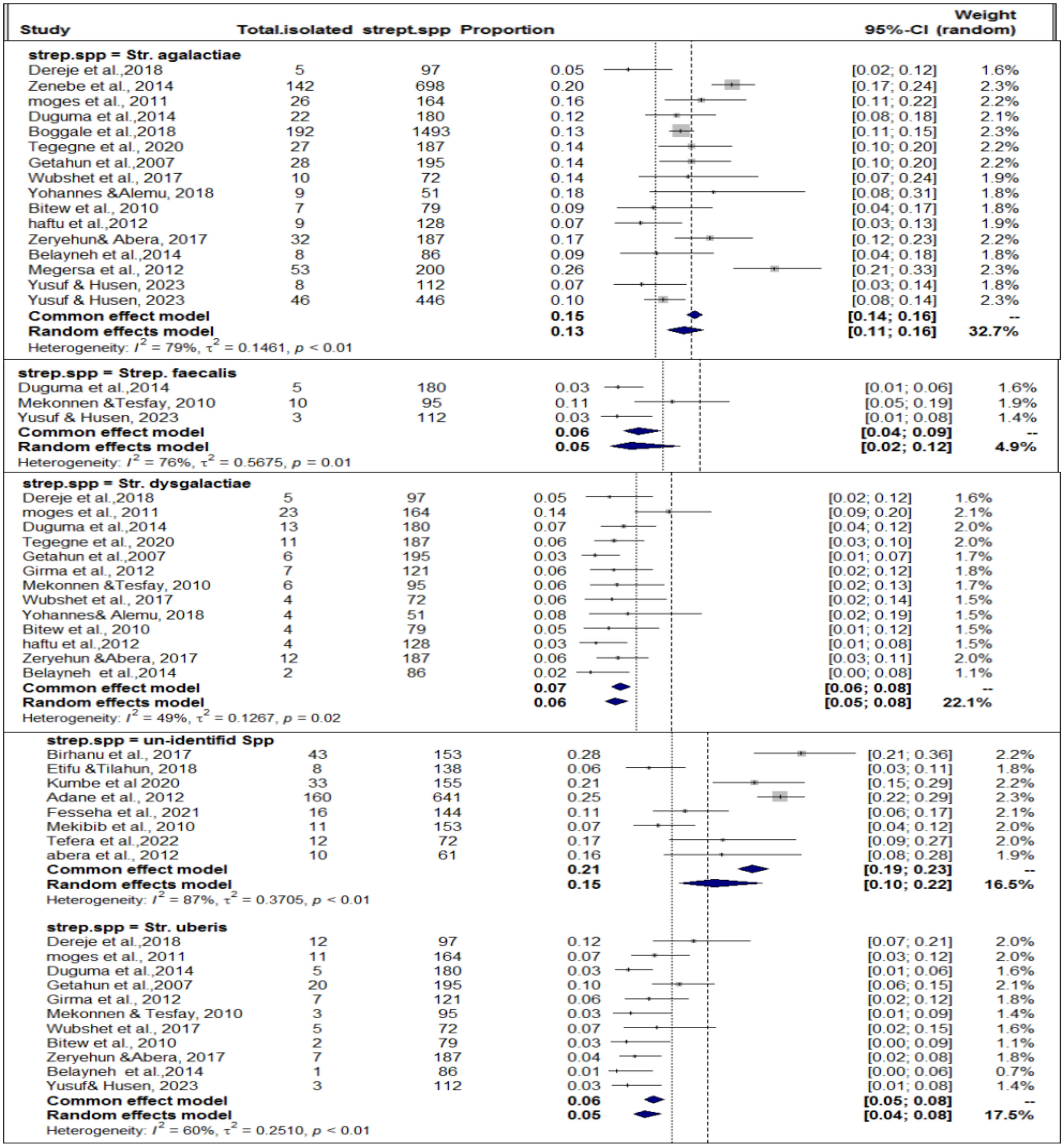

Figure 4 Subgroup analysis of the proportion of mastitis-associated *Sterptococcus* by type of species
